# Early Childhood Adversity Predicts Risk of Family Caregiver Suicidal Ideation:Roles of Neuroticism and Self-Compassion

**DOI:** 10.21203/rs.3.rs-4803419/v1

**Published:** 2024-09-17

**Authors:** Felipe A. Jain, Paulina Gutierrez-Ramirez, Miranda Zea, Olivia I. Okereke, Kimberly A. Van Orden, Paola Pedrelli, Ana-Maria Vranceanu, Kimberly Dueck, Aderonke Pederson, Liliana A. Ramirez Gomez

**Affiliations:** Depression Clinical and Research Program, Department of Psychiatry, Massachusetts General Hospital, Boston, MA; Department of Psychiatry, Massachusetts General Hospital and Harvard Medical School, Boston, MA; Harvard-MIT Division of Health Sciences and Technology, Boston, MA; Depression Clinical and Research Program, Department of Psychiatry, Massachusetts General Hospital, Boston, MA; Depression Clinical and Research Program, Department of Psychiatry, Massachusetts General Hospital, Boston, MA; Department of Psychiatry, Massachusetts General Hospital and Harvard Medical School, Boston, MA; Department of Epidemiology, Harvard-T.H. Chan School of Public Health, Boston, MA; Department of Psychiatry, University of Rochester, Rochester, NY; Depression Clinical and Research Program, Department of Psychiatry, Massachusetts General Hospital, Boston, MA; Department of Psychiatry, Massachusetts General Hospital and Harvard Medical School, Boston, MA; Department of Psychiatry, Massachusetts General Hospital and Harvard Medical School, Boston, MA; Center for Health Outcomes and Interdisciplinary Research, Department of Psychiatry, Massachusetts General Hospital, Boston, MA; Depression Clinical and Research Program, Department of Psychiatry, Massachusetts General Hospital, Boston, MA; Depression Clinical and Research Program, Department of Psychiatry, Massachusetts General Hospital, Boston, MA; Department of Psychiatry, Massachusetts General Hospital and Harvard Medical School, Boston, MA; Memory Disorders Division, Department of Neurology, Massachusetts General Hospital, Boston, MA; Department of Neurology, Harvard Medical School, Boston, MA

## Abstract

**Background:**

Despite high rates of family caregiver suicidal ideation (SI), little is known about its relationship with childhood adversity. Those with a history of adverse childhood experiences (ACEs) have been shown to have higher neuroticism, lower self-compassion, and higher rates of late life mental health disorders. Caregiving for a family member with dementia may pose a particular challenge for those with ACEs.

**Methods:**

In a secondary analysis of 81 family caregivers of people living with dementia enrolled in clinical trials, we undertook a cross-sectional baseline analysis of the association between childhood adversity, measured with the ACE questionnaire, and self-reported suicidal ideation (SI). We further assessed whether the relationship between ACE and SI was mediated by neuroticism and self-compassion.

**Results:**

18 caregivers self-reported SI (22%). 89% of caregivers with SI reported childhood adversity (ACE > 0), versus 63% of those without SI (p=.04). The relative risk of SI was 3.6x higher in those with childhood adversity than in those without (p=.04), and for those with a specific history childhood abuse, the relative risk of SI was 3.4x higher (p=.005). Neuroticism and self-compassion mediated the relationship between ACE and SI (p<.05), with neuroticism strengthening the association and self-compassion weakening it.

**Conclusions:**

The association of SI with history of childhood adversity is high in family caregivers. Whereas elevated neuroticism might be one mechanism linking ACEs and SI, training self-compassion is a promising target for reducing SI. The phenotypic relationship between childhood adversity and SI in family caregivers should be further explored in larger samples, and could represent a new treatment target to improve the efficacy of therapies on caregiver emotional symptoms.

## 1. Introduction

Family caregivers of people living with a chronic illness such as dementia exhibit high rates of suicidal ideation (SI).^1^ In 2021, the United States Centers for Disease Control reported serious suicidal ideation in the past month in approximately 30% of family caregivers, and a three-fold increased risk of SI, specifically for family caregivers of those with cognitive disorders.^2^ Elevated caregiver SI has also been reported in several countries in smaller studies across the globe.^3–7^

Caregiving and volunteering involve prosocial activities and contributing to others. However, while volunteering is linked to better mental health and reduced risk for suicide,^8^ caregiving is not associated with reduced risk for suicide,^8^ and instead is linked to poorer mental health and increased likelihood of suicide ideation. The majority of caregivers who think about suicide will not attempt or die by suicide, however, the presence of suicide ideation is one of the strongest risk factors for subsequent suicide attempts and deaths.^9^ SI in family caregivers is also associated with poor outcomes in individuals receiving care, some tragic. One study documented associations of caregiver SI with higher rates of abusive behaviors.^3^ Newspaper reports in the United States and Korea have identified cases of homicide-suicide.^10,11^ There is likely underreporting of these issues due to very limited data collection on SI and SI-related outcomes in caregivers.

Factors associated with family caregiver SI include caregiver burden,^3,5,7^ depression,^4,5,12,13^ anxiety,^3–5,13^ and psychological stress.^7,13,14^ External factors to the caregiver associated with SI include financial stressors^5,7^ and lack of family support.^4^ These factors have all been identified as occuring contemporaneously with actively caregiving. However, less is known about how developmental and personality factors over the lifespan might influence the emergence of SI in the caregiving situation.

The life course theory posits that adverse events that occur early in life, such as childhood trauma, influence the manifestation of late-life mood and behavioral symptoms.^15^ Adverse childhood experiences (ACEs) including abuse, neglect, and household dysfunction, may result in abnormal psychological development.^16^ ACEs have been identified as risk factors for late-life mood problems^17–20^ and death by suicide.^21^ Among family caregivers, those caring for parents who abused or neglected them as children were found to exhibit higher depressive symptoms.^22^

A potential mechanism linking ACEs to late life mood disorders and suicide risk is the lifelong development of neuroticism, a personality trait characterized by a predisposition to negative affect,^23^ particularly in the setting of stressful life events.^24^ Adults with a history of exposure to childhood maltreatment demonstrate a higher degree of neuroticism than those without.^25–27^ Neuroticism has also been found to mediate the relationship between ACEs and later life depressive symptoms, demonstrating the impact of ACEs on mental health across the life course.^28,29^

Spousal caregivers who have high levels of neuroticism demonstrate higher caregiver strain and depressive symptoms.^30^ In caregivers of people living with dementia, higher levels of neuroticism have been associated with depressive symptoms^31,32^ and caregiver burden.^33^ Mediators of the relationship between neuroticism and poor mental health include perceived stress and healthful behaviors,^31^ and caregiver strain and self-efficacy.^34^ Neuroticism has been found to moderate the relationship between caregiver burden and anxiety symptoms: caregivers with higher levels of neuroticism evinced higher levels of anxiety than those with lower neuroticism at the same level of caregiver burden.^35^ Neuroticism has also been associated with higher physical and psychological abuse of care recipients.^36^

Positive psychological traits might be protective against consequences of adverse childhood experiences. Self-compassion is a multi-component construct that describes an individual’s capacity for self-kindness, their ability to situate stressors and negative experiences as part of a common human experience, and to focus on negative experiences without self-judgment.^37^ Self-compassion focuses on “soothing and comforting the ‘self’” in the context of painful experiences.^38^ Systematic meta-analysis of studies in young and middle aged adults has found that self-compassion is negatively correlated with SI and suicidal behaviors with a moderate effect size.^39^ In stressful situations where perceived failings or experiences of shame might underlie SI, self-compassion might facilitate adaptive coping, self-soothing, and healthy behaviors.

In this study, we aimed to determine the cross-sectional relationship between ACEs and SI in a predominantly older adults sample of family caregivers of people living with dementia who sought care in clinical trials aimed at improving mood. We further aimed to identify possible psychological mechanisms linking ACEs to SI, with a focus on neuroticism and self-compassion. We hypothesized that those with ACEs would be more likely to report SI, and that neuroticism would be associated with SI and would in part account for the relationship between ACE and SI. We also hypothesized that self-compassion would be a protective factor negatively associated with SI and also mediating the relationship between ACE and SI in a direction opposite to that of neuroticism.

## 2. Methods

Participants were identified from a convenience sample of individuals who self-identified as the primary family caregiver of a person living with dementia and were enrolled in one of two pilot clinical trials aimed at improving mood between 2015 and 2017 at the University of California, San Francisco (UCSF, Institutional Review Board Protocol #16–20163). The focus of this report is on baseline data collected prior to any intervention. The first trial was an in-person study of 4-week mentalizing imagery therapy (MIT) for caregivers; the second trial comprised an add-on feasibility study of a smartphone MIT application for caregivers living far from the study site or who could not participate in person for other reasons (e.g. transportation, time); outcomes from both trials have been previously reported.^13,40,41^ Recruitment methods involved distributing flyers at community centers and events, and sending direct mail to patients living with dementia to share with their caregivers.

Inclusion criteria were reporting being the primary family member responsible for the care of a relative with dementia, being 40 years of age or older, English language fluency, and ability to give informed consent. Exclusion criteria included ideas of harming the relative with dementia, adult protective services report on file, primary psychiatric disorder other than unipolar major depression, caregiver cognitive impairment, unstable medical illness or planned surgery, and current drug or alcohol use disorder.

All participants provided written, informed consent. Following consent, participants filled out self-report questionnaires. 50 of the participants also completed onsite caregiver demographic interviews (e.g. length of time caregiving, number of hours per week, relative with dementia)^41^; this data was unavailable from the other 31 participants because they were evaluated as part of a remote add-on mobile application study for which limited funding was available and for which participants did not come to the study site. If participants were identified by baseline assessments as having active suicidal ideation, they could not proceed to the clinical trial portion of the study and instead were evaluated by a licensed psychiatrist and referred to standard of care treatment.

### Measures:

The 17-item Adverse Childhood Experiences (ACEs) scale was used to measure childhood abuse, neglect, and household dysfunction.^42,43^ Similar to prior analyses, three subscales were computed: abuse (items assessing physical, emotional, or sexual abuse), neglect (physical, emotional), and household dysfunction (parents separated or divorced, mother abused or threatened, alcohol or substance use in home, mental illness in household, family member in prison).^43^ Self-reported SI in the past seven days was drawn from item #12 of the Quick Inventory of Depressive Symptoms Self-Report, “Thoughts of Death or Suicide”.^44^ Response choices for this item include 0=“I do not think of suicide or death.”, 1=“I feel that life is empty or wonder if it’s worth living.”, 2=“I think of suicide or death several times a week for several minutes.”, 3=“I think of suicide or death several times a day in some detail, or I have made specific plans for suicide or have actually tried to take my life.” Depressive symptoms without SI were separately computed based on the total QIDS score minus the SI item. Caregiver burden was calculated as the total score of the Zarit Burden Interview.^45^ Neuroticism was estimated with a two-item subscale from the Ten Item Personality Inventory, which is rated from 1 to 7 on a Likert scale (minimum score 2, maximum score 14, higher indicating more neuroticism).^46,47^ Self-compassion was assessed with the Self Compassion Short Form.^48^

### Statistical analysis:

All statistical analyses were performed with R version 4.3.2.^49^ To determine whether participants had experienced maltreatment, total scores were binarized for the ACE or ACE subscales (0 for none, 1 for 1 or more ACE), and these binary scores were used in all subsequent analyses. For SI, response of 1 or higher was coded as presence of SI, whereas a score of 0 indicated no SI. Total scores were calculated for the self-compassion, neuroticism, caregiver burden, and other depressive symptoms. Non-parametric Spearman correlation among all variables was performed. 2×2 tables were constructed on the presence/absence of SI and ACE. Relative risk ratios were determined with 95% confidence intervals using package “epitools” in R.^50^ Model significance was assessed by chi square.

Mediation analyses were performed on the ACE measure with highest statistical significance in a 4-step process. First, according to the procedures of Baron and Kenny,^51^ a pathway analysis was performed. The direct pathway from the ACE measure to SI (binary outcome) was computed using logistic regression. Second, the pathway from the ACE measure to the mediator (ordinal variable) was calculated using linear regression. Third, logistic regression was used to compute the pathway from the ACE measure to SI, adjusting for the effect of the mediator. Finally, to estimate the proportion of the variance of the direct pathway accounted for by the mediator, mediation analyses with bootstrapping 1000 repetitions was performed with R package “mediation”,^52^ using the presence/absence of the ACE measure as the predictor, neuroticism or self-compassion as the mediator, and the presence/absence of SI as the outcome.

## 3. Results

### 3.1 Demographics and SI prevalence

The overall sample was 80% female. Mean age was 64 (9 standard deviation) years. Prevalence of participant race was 79% White, 14% Asian / Pacific Islander, 5% Black / African American, and 2% more than one race; regarding ethnicity, 4% were Hispanic. 15 participants reported a 1 on the QIDS SI measure and 3 participants reported a 2; none reported a 3.

Of the 50 caregivers for whom specific caregiving data was available, 36% were caring for a parent, 46% for a spouse, and 18% for another relative (e.g. aunt, uncle, sibling). 68% were living with the person with dementia. They had been caring for an average of 5 (SD 3) years, and on average were spending 62 hours (SD 47) weekly on caregiving responsibilities.

### 3.2 Correlation among baseline measures (Table 2)

Female sex was associated with higher rates of having at least one ACE and a history of childhood abuse specifically, increased neuroticism, and lower self-compassion (p < .05 for all). Older age was associated with lower likelihood of having at least one ACE and lower childhood household dysfunction specifically (p < .05). Neuroticism was strongly negatively associated with self-compassion (p < .001), and was associated with other depressive symptoms (p < .001), caregiver burden (p < .001), history of abuse (p < .05), neglect (p < .05), and SI (p < .01). Higher self-compassion was associated with lower reported depressive symptoms (p < .001), caregiver burden (p < .001), childhood abuse (p < .05) and household dysfunction (p < .05), and lower SI (p < .01). Depressive symptoms were associated with a childhood history of any ACE (p < .05), neglect (p < .05), and SI (p < .05). Caregiver burden was associated with a childhood history of neglect (p < .01) and with SI (p < .01). The three ACE subtypes were significantly intercorrelated (p < .001), but among them, only a history of childhood abuse was associated with SI (p < .01).

### 3.3 Relative risk ratios for childhood maltreatment and SI (Table 2)

History of any ACE was associated with a relative risk of 3.6 for SI (p = .04). This effect was largely driven by a history of childhood abuse, for which the relative risk of SI was 3.4 (p = .005). Neither history of childhood neglect, for which the relative risk ratio was 1.5 (p = .3), nor household dysfunction, for which the relative risk ratio was 1.1 (p = .8), were associated with SI.

### 3.4 Mediation analyses

#### 3.4.A Mediation modeling

**Figure 1:**
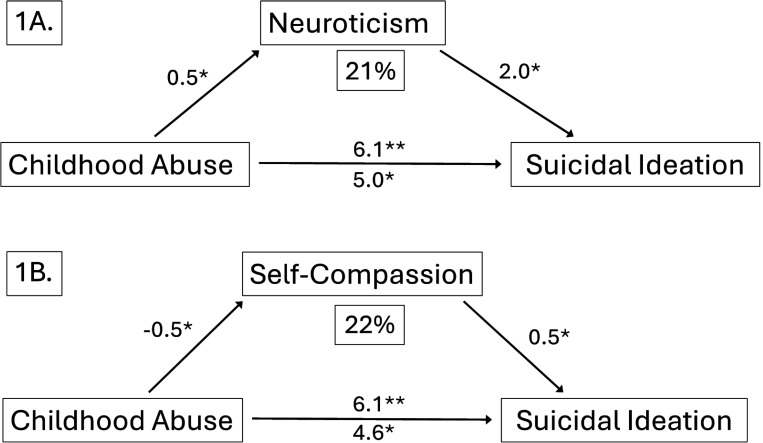
Mediation model. A. Partial mediation of suicidal ideation by neuroticism. B. Partial mediation of suicidal ideation by self-compassion. The percent in the box beneath neuroticism and self-compassion labels indicates the proportion of the direct pathway each trait mediates. Coefficients from abuse to neuroticism and self-compassion indicate normalized regression coefficient. All coefficients leading to suicidal ideation show odds ratios as inferred from the logistic regression model. For the pathway from abuse to suicidal ideation, the partial odds ratio from the logistic regression model adjusting for the mediator is shown beneath the arrow.

Because the most significant risk for SI was found in caregivers with a history of childhood abuse, we focused on understanding mediators that linked childhood abuse to SI. In model 1 (Fig. 1A), neuroticism was tested as a mediator. Pathway analysis demonstrated that there was a significant direct effect between the presence of any childhood abuse and SI (p < .01), which was partially mediated by neuroticism (p < .05). Mediation analyses with bootstrapping of Model 1 verified that neuroticism mediated the effect of abuse on SI (p = .02), with a proportion mediated of 21%.

In model 2, self-compassion was tested as a mediator (Fig. 1B). Pathway analysis found that the relationship between childhood abuse and SI was also partially mediated by self-compassion (p < .05). Mediation analysis with bootstrapping of model 2 found that self-compassion mediated the effect of abuse on SI (p = .04), with a proportion mediated of 22%.

## Discussion

The major finding of this investigation is that a history of childhood maltreatment, especially abuse, is one experience that may predispose family caregivers to SI. To our knowledge, this is the first finding of a relationship between early life childhood experiences and SI in caregivers. Previous work cross-sectionally identified factors such as depression, anxiety, caregiver burden, and stress, all contemporaneous with SI. However, a relationship between adverse childhood experiences and family caregiver SI has not been described. Our findings provide support for the life course model of early childhood experiences impacting late-life mental health,^15^ including thoughts of suicide. These results illustrate one pathway whereby enduring risk factors such as childhood abuse can impact suicide risk in later life via the development of personality characteristics and ways of interacting with the world that decrease the ability to cope optimally with stressors that occur more commonly in later life, including caregiving.

Our results further characterize the first psychological mediators linking childhood abuse to caregiver SI. Specifically, a personality factor related to poor emotion regulation, neuroticism, mediated the pathway from childhood abuse to caregiver SI. In adolescents and younger adults, neuroticism has been found to mediate associations between ACEs and suicidal behaviors.^53,54^ Our work extends these findings to a predominantly older adult sample. Neuroticism might be particularly maladaptive for caregivers due to the amplification of the emotional response to caregiver stressors. Caregiver stressors are chronic and cumulative,^55^ and those with a high degree of neuroticism may find themselves repeatedly triggered and predisposed to overwhelmingness, anxiety, and hopelessness. The high degree of negative affectivity in caregivers with high neuroticism may also negatively impact their ability to provide care for their relative.

Data overall are limited on effective mental health therapies for older adults with a history of childhood adversity, not to mention caregivers.^56^ These mediation analyses provided potential identification of a trainable treatment target: self-compassion. Self-compassion reduced the association between childhood abuse and SI. Self-compassion is a construct that measures the degree of self-kindness and ability to contextualize distress as part of a shared human experience. Several therapies may train self-compassion, including mindful self-compassion^57^ and Mentalizing Imagery Therapy (MIT).^41^ In a partially overlapping sample of the data from this study, we recently reported that a 4-week MIT program reduced family dementia caregiver SI relative to psychosocial support intervention. This was the first evidence from a randomized controlled trial of a differential therapeutic effect on SI in caregivers.^13^ We also previously showed that MIT boosted self-compassion more than psychoeducation and support.^41^ Future work in larger samples should identify whether longitudinal increases in self-compassion mediate the reduction in SI with MIT and other therapeutic approaches.

Among ACEs, a history of childhood abuse was strongly related to SI but not household dysfunction or neglect. These results do not indicate, however, that neglect may not be related to other caregiver outcomes, such as forgoing self-care, such as exercise or doctor visits, and these outcomes should be studied in additional research. Indeed, neglect was the only ACE subscale correlated with caregiver burden, suggesting that those without a childhood experience of being adequately cared for emotionally or physically might also find the challenge of caring for another particularly burdensome.

Limitations of this work include a lack of inclusion of participants with thoughts of violence or an adult protective services report on file. Given the comorbidity between abusive behaviors toward a relative with dementia and SI,^3^ this population should be included in future research. We also were unable to address whether specific caregiving characteristics (e.g., caring for a perpetrating parent) might have moderated the results. The cross-sectional design poses a limitation for making study inferences; however, we recognize that caregiving in itself is unlikely to increase neuroticism or reduces self-compassion and, thus, there is a face validity to the hypothesized path of associations. Our measure of self-report SI utilized a single item from a validated depression rating scale; future research should use more comprehensive measures of SI and confirm these findings in larger samples. Evaluation of ACEs is limited by retrospective reporting, which could be influenced by recall bias. Racial and ethnic diversity was limited: although 21% of participants came from groups underrepresented in research, there was a relative overrepresentation of those from Asian background (14%), in comparison to Black (5%) and Hispanic or Latino (4%). Finally, this was a convenience sample of caregivers seeking treatment and not a representative sample of the population.

Although we have demonstrated an association between childhood adversity and SI, the risk of suicide attempts or death by suicide is unclear in caregivers due to a dearth of adequately powered studies. Data capture from this population is complicated by the fact that caregivers with SI might also seek to abandon the caregiver role (e.g. by institutionalizing the relative with chronic illness, as has been found for relatives of caregivers with high levels of psychological distress).^58^ The severity of SI as a symptom behooves further investigation and increased study in both observational and treatment trials. Moreover, we suggest that interventions for caregivers should consider the extent to which childhood trauma might inform the clinical presentation. Further, there is a need for the development of personalized caregiver programs that focus not just on managing stressors but on increasing self-compassion and accounting for life-course factors.

## Figures and Tables

**Table 1.** Correlation among variables

	Sex (F)	Age	Caregiver Burden	Depressive Symptoms	Neuroticism	Self-compassion	Any ACE	Abuse	Household dysfunction	Neglect	Suicidal ideation
Sex (F)	1										
Age	−0.13	1									
Caregiver Burden	0.33**	−0.26*	1								
Depressive Symptoms	0.22	−0.04	0.43***	1							
Neuroticism	0.24*	−0.15	0.44***	0.51***	1						
Self-compassion	−0.22*	0.21	−0.56***	−0.39***	−0.69	1					
Any ACE	0.27*	−0.22*	0.21	0.23*	0.21	−0.07	1				
Abuse	0.25*	−0.06	0.18	0.21	0.24*	−0.25*	0.58***	1			
Household dysfunction	0.15	−0.23*	0.12	0.15	0.03	−0.04	0.79***	0.44***	1		
Neglect	0.13	−0.03	0.35**	0.26*	0.33**	−0.29**	0.38***	0.37***	0.2	1	
Suicidal ideation	0.18	−0.04	0.31**	0.24*	0.33**	−0.37***	0.23*	0.31**	0.03	0.11	1

ACE = adverse childhood experiences,

SI = suicidal ideation,

* p < .05,

** p < .01,

*** p < .001

**Table 2.** Relative risk ratio for adverse childhood experiences and SI (n = 81)

	Incidence Overall	Incidence No SI	Incidence SI	Relative risk ratio [95% CI]	Chi square p-value
Any ACE	56 (69%)	40 (63%)	16 (89%)	3.6 [1.3, Inf]	0.04
Abuse	35 (43%)	22 (35%)	13 (72%)	3.4 [1.5, 14.5]	0.005
Neglect	20 (25%)	14 (22%)	6 (33%)	1.5 [0.5, 3.4]	0.3
Household dysfunction	47 (58%)	36 (57%)	11 (61%)	1.1 [0.5, 3.1]	0.8

ACE = adverse childhood experiences, Inf = infinite, i.e. formula did not converge; SI = suicidal ideation

## References

[1] O’Dwyer ST, Janssens A, Sansom A (2021) Suicidality in family caregivers of people with long-term illnesses and disabilities: A scoping review. Compr Psychiatry 110:152261 34332205 10.1016/j.comppsych.2021.152261

[2] Czeisler M, Rohan EA, Melillo S (2021) Mental Health Among Parents of Children Aged < 18 Years and Unpaid Caregivers of Adults During the COVID-19 Pandemic — United States, December 2020 and February–March 2021. MMWR Morb Mortal Wkly Rep 70:879–887 34138835 10.15585/mmwr.mm7024a3 PMC8220951

[3] Teasdale-Dubé A, Viau-Quesnel C, Lapierre S (2024) Suicidal Ideation in Canadian Family Caregivers for a Person with Dementia: A Portrait of the Situation. Can J Aging Rev Can Vieil 1–8. 10.1017/S0714980824000011 38317578

[4] Joling KJ, O’Dwyer ST, Hertogh CMPM, van Hout HPJ (2018) The occurrence and persistence of thoughts of suicide, self-harm and death in family caregivers of people with dementia: a longitudinal data analysis over 2 years. Int J Geriatr Psychiatry 33:263–270 28379646 10.1002/gps.4708 PMC5811919

[5] O’Dwyer ST, Moyle W, Zimmer-Gembeck M, De Leo D (2016) Suicidal ideation in family carers of people with dementia. Aging Ment Health 20:222–230 26161825 10.1080/13607863.2015.1063109

[6] Du N-H, Han S-J (2018) Factors Affecting the Suicidal Ideation in Spouse Caregivers of the Elderly with Dementia Living in the Community. J Korea Converg Soc 9:241–250

[7] Dos Santos Treichel CA, da Jardim R, Prado Kantorski VM (2019) Guimarães Lima, M. Prevalence and factors associated with suicidal ideation among family caregivers of people with mental disorders. J Clin Nurs 28:3470–3477 31162868 10.1111/jocn.14938

[8] Rosato M, Tseliou F, Wright DM, Maguire A, O’Reilly D (2019) Are volunteering and caregiving associated with suicide risk? A Census-based longitudinal study. BMC Psychiatry 19:296 31601191 10.1186/s12888-019-2255-8 PMC6788116

[9] Franklin JC, Ribeiro JD, Fox KR (2017) Risk factors for suicidal thoughts and behaviors: A meta-analysis of 50 years of research. Psychol Bull 143:187–232 27841450 10.1037/bul0000084

[10] Malphurs JE, Cohen D (2005) A statewide case-control study of spousal homicide-suicide in older persons. Am J Geriatr Psychiatry Off J Am Assoc Geriatr Psychiatry 13:211–217 10.1176/appi.ajgp.13.3.211 15728752

[11] Kim W (2014) Analysis of newspaper articles on suicides and homicides in family members with dementia. J Health Soc Welf 34:219–246

[12] Kim HJ, Kehoe P, Gibbs LM, Lee J-A (2019) Caregiving Experience of Dementia among Korean American Family Caregivers. Issues Ment Health Nurs 40:158–165 30620625 10.1080/01612840.2018.1534909 PMC6445682

[13] Madarasmi S, Gutierrez-Ramirez P, Barsoum N (2024) Family dementia caregivers with suicidal ideation improve with mentalizing imagery therapy: Results from a pilot study. J Affect Disord Rep 16:100721 38737194 10.1016/j.jadr.2024.100721 PMC11086673

[14] Jeong H-C (2017) A Study on the Effect of Elderly Dementia Caregiver’s Stress to their Suicidal Ideation -Mediating Effect of Self-efficacy-. J Korea Contents Assoc 17:167–182

[15] Pearlin LI, Schieman S, Fazio EM, Meersman SC, Stress (2005) Health, and the Life Course: Some Conceptual Perspectives. J Health Soc Behav 46:205–219 16028458 10.1177/002214650504600206

[16] Hughes K, Bellis MA, Hardcastle KA (2017) The effect of multiple adverse childhood experiences on health: a systematic review and meta-analysis. Lancet Public Health 2:e356–e366 29253477 10.1016/S2468-2667(17)30118-4

[17] Selous C, Kelly-Irving M, Maughan B (2020) Adverse childhood experiences and adult mood problems: evidence from a five-decade prospective birth cohort. Psychol Med 50:2444–2451 31583986 10.1017/S003329171900271X

[18] Dagnino P, Ugarte MJ, Morales F (2020) Risk Factors for Adult Depression: Adverse Childhood Experiences and Personality Functioning. Front Psychol 11 10.3389/fpsyg.2020.594698 PMC7762330 33362658

[19] Cheong EV, Sinnott C, Dahly D, Kearney PM (2017) Adverse childhood experiences (ACEs) and later-life depression: perceived social support as a potential protective factor. BMJ Open 7:e013228 10.1136/bmjopen-2016-013228 PMC5588961 28864684

[20] Raposo SM, Mackenzie CS, Henriksen CA, Afifi TO (2014) Time Does Not Heal All Wounds: Older Adults Who Experienced Childhood Adversities Have Higher Odds of Mood, Anxiety, and Personality Disorders. Am J Geriatr Psychiatry 22:1241–1250 24012227 10.1016/j.jagp.2013.04.009

[21] Favril L, Yu R, Uyar A, Sharpe M, Fazel S (2022) Risk factors for suicide in adults: systematic review and meta-analysis of psychological autopsy studies. BMJ Ment Health 25:148–155 10.1136/ebmental-2022-300549 PMC9685708 36162975

[22] Kong J, Moorman SM (2015) Caring for My Abuser: Childhood Maltreatment and Caregiver Depression. Gerontologist 55:656–666 24265506 10.1093/geront/gnt136

[23] Costa PT, McCrae RR (1985) Hypochondriasis, neuroticism, and aging: When are somatic complaints unfounded? Am Psychol 40:19–28 3977166 10.1037//0003-066x.40.1.19

[24] Lahey BB (2009) Public health significance of neuroticism. Am Psychol 64:241–256 19449983 10.1037/a0015309 PMC2792076

[25] Fletcher JM, Schurer S (2017) Origins of Adulthood Personality: The Role of Adverse Childhood Experiences. BE J Econ Anal Policy 17 10.1515/bejeap-2015-0212 PMC6063370 30057657

[26] Sachs-Ericsson NJ, Rushing NC, Stanley IH, Sheffler J (2016) In my end is my beginning: developmental trajectories of adverse childhood experiences to late-life suicide. Aging Ment Health 20:139–165 26264208 10.1080/13607863.2015.1063107

[27] Grusnick JM, Garacci E, Eiler C, Williams JS, Egede LE (2020) The association between adverse childhood experiences and personality, emotions and affect: Does number and type of experiences matter? J Res Personal 85:103908 10.1016/j.jrp.2019.103908 PMC7453784 32863469

[28] Lee M-A, Song R (2017) Childhood abuse, personality traits, and depressive symptoms in adulthood. Child Abuse Negl 65:194–203 28189957 10.1016/j.chiabu.2017.02.009

[29] Li Y, Cheng L, Guo L (2023) Mediating role of personality traits in the association between multi-dimensional adverse childhood experiences and depressive symptoms among older adults: A 9-year prospective cohort study. J Affect Disord 331:167–174 36963513 10.1016/j.jad.2023.03.067

[30] Bookwala J&, Schulz R (1998) The Role of Neuroticism and Mastery in Spouse Caregivers’ Assessment of and Response to a Contextual Stressor. J Gerontol Ser B 53B:P155–P164 10.1093/geronb/53b.3.p155 9602831

[31] Gallant MP, Connell CM (2003) Neuroticism and depressive symptoms among spouse caregivers: Do health behaviors mediate this relationship? Psychol Aging 18:587–592 14518817 10.1037/0882-7974.18.3.587

[32] González-Abraldes I, Millán-Calenti JC, Lorenzo-López L, Maseda A (2013) The influence of neuroticism and extraversion on the perceived burden of dementia caregivers: An exploratory study. Arch Gerontol Geriatr 56:91–95 22906469 10.1016/j.archger.2012.07.011

[33] Shurgot GR, Knight BG (2005) Influence of Neuroticism, Ethnicity, Familism, and Social Support on Perceived Burden in Dementia Caregivers: Pilot Test of the Transactional Stress and Social Support Model. J Gerontol Ser B 60:P331–P334 10.1093/geronb/60.6.p331 16260707

[34] Löckenhoff CE, Duberstein PR, Friedman B, Costa PT Jr. (2011) Five-factor personality traits and subjective health among caregivers: The role of caregiver strain and self-efficacy. Psychol Aging 26:592–604 21417534 10.1037/a0022209 PMC3168724

[35] Yang XY, Morton P, Yang F, Fang B (2022) The Moderation Role of Neuroticism for Anxiety among Burdened Dementia Caregivers: A Study on Care Giver-Recipient Dyads. J Gerontol Soc Work 65:692–710 34985409 10.1080/01634372.2021.2019164

[36] Fang B, Yan E, Yang X, Pei Y (2021) Association between Caregiver Neurotic Personality Trait and Elder Abuse: Investigating the Moderating Role of Change in the Level of Caregiver Perceived Burden. Gerontology 67:243–254 33454703 10.1159/000512238

[37] Neff KD (2009) The Role of Self-Compassion in Development: A Healthier Way to Relate to Oneself. Hum Dev 52:211–214 22479080 10.1159/000215071 PMC2790748

[38] Germer CK, Neff KD (2013) Self-compassion in clinical practice. J Clin Psychol 69:856–867 23775511 10.1002/jclp.22021

[39] Per M, Schmelefske E, Brophy K, Austin SB, Khoury B, Mindfulness (2022) Self-compassion, Self-injury, and Suicidal thoughts and Behaviors: a Correlational Meta-analysis. Mindfulness 13:821–842

[40] Sikder A, Yang F, Schafer R (2019) Mentalizing Imagery Therapy Mobile App to Enhance the Mood of Family Dementia Caregivers: Feasibility and Limited Efficacy Testing. JMIR Aging 2:e12850 31518275 10.2196/12850 PMC6715046

[41] Jain FA, Chernyak S, Nickerson LD (2022) 4-week Mentalizing Imagery Therapy for family dementia caregivers: A randomized controlled trial with neural circuit changes. Psychother Psychosom 91:180–189 35287133 10.1159/000521950 PMC9064903

[42] Felitti VJ, Anda RF, Nordenberg D (1998) Relationship of Childhood Abuse and Household Dysfunction to Many of the Leading Causes of Death in Adults: The Adverse Childhood Experiences (ACE) Study. Am J Prev Med 14:245–258 9635069 10.1016/s0749-3797(98)00017-8

[43] Dube SR, Felitti VJ, Dong M (2003) Childhood Abuse, Neglect, and Household Dysfunction and the Risk of Illicit Drug Use: The Adverse Childhood Experiences Study. Pediatrics 111:564–572 12612237 10.1542/peds.111.3.564

[44] Rush AJ, Trivedi MH, Ibrahim HM (2003) The 16-Item quick inventory of depressive symptomatology (QIDS), clinician rating (QIDS-C), and self-report (QIDS-SR): a psychometric evaluation in patients with chronic major depression. Biol Psychiatry 54:573–583 12946886 10.1016/s0006-3223(02)01866-8

[45] Bédard M, Molloy DW, Squire L (2001) The Zarit Burden Interview. Gerontologist 41:652–657 11574710 10.1093/geront/41.5.652

[46] Gosling SD, Rentfrow PJ, Swann WB (2012) Ten-Item Personality Inventory. 10.1037/t07016-000

[47] Ehrhart MG, Ehrhart KH, Roesch SC (2009) Testing the latent factor structure and construct validity of the Ten-Item Personality Inventory. Personal Individ Differ 47:900–905

[48] Neff KD (2003) The Development and Validation of a Scale to Measure Self-Compassion. Self Identity 2:223–250

[49] R Core Team (2018) R: A language and environment for statistical computing. R Foundation for Statistical Computing

[50] Aragon TJ, Fay MP, Wollschlaeger D, Omidpanah A (2012) EpiTools: R package for epidemiologic data and graphics. v0.5–10.1

[51] Baron RM, Kenny DA (1986) The moderator–mediator variable distinction in social psychological research: Conceptual, strategic, and statistical considerations. J Pers Soc Psychol 51:1173–1182 3806354 10.1037//0022-3514.51.6.1173

[52] Tingley D, Yamamoto T, Hirose K, Keele L, Imai K, Mediation (2014) R package for causal mediation analysis. J Stat Softw 59

[53] Jirakran K, Vasupanrajit A, Tunvirachaisakul C, Maes M (2023) The effects of adverse childhood experiences on depression and suicidal behaviors are partially mediated by neuroticism: A subclinical manifestation of major depression. Front Psychiatry 14 10.3389/fpsyt.2023.1158036 PMC10169750 37181874

[54] Mozafari S, Bahadivand AH, Khodarahimi S, Mazraeh N, Rahimianbougar M (2023) The role of adverse childhood experiences and defense mechanisms on suicidal ideation and social dysfunction. Curr Psychol. 10.1007/s12144-023-04742-7

[55] Pearlin LI, Mullan JT, Semple SJ, Skaff MM (1990) Caregiving and the stress process: an overview of concepts and their measures. Gerontologist 30:583–594 2276631 10.1093/geront/30.5.583

[56] Ishikawa RZ, Ander I, Popescu DL, Vyas CM, Okereke OI (2023) Child Maltreatment Among Older Adults: A Narrative Review of Psychotherapeutic Interventions and Clinical Considerations. Clin Gerontol 1–15. 10.1080/07317115.2023.2219671 37254789

[57] Neff KD, Germer CKA, Pilot Study (2013) Randomized Controlled Trial of the Mindful Self-Compassion Program. J Clin Psychol 69:28–44 23070875 10.1002/jclp.21923

[58] Gaugler JE, Yu F, Krichbaum K, Wyman JF (2009) Predictors of nursing home admission for persons with dementia. Med Care 47:191–198 19169120 10.1097/MLR.0b013e31818457ce

